# Validation of methods for prediction of clinical output levels of active middle ear implants from measurements in human cadaveric ears

**DOI:** 10.1038/s41598-017-16107-9

**Published:** 2017-11-20

**Authors:** Martin Grossöhmichen, Bernd Waldmann, Rolf Salcher, Nils Prenzler, Thomas Lenarz, Hannes Maier

**Affiliations:** 1 Department of Otolaryngology and Institute of Audioneurotechnology (VIANNA), Hannover Medical School, 30625 Hannover, Germany; 2 DFG Cluster of Excellence, Hearing4all, Germany; 3 Cochlear Deutschland GmbH & Co. KG, 30625 Hannover, Germany

## Abstract

Today, the standard method to predict output levels of active middle ear implants (AMEIs) before clinical data are available is stapes vibration measurement in human cadaveric ears, according to ASTM standard F2504-05. Although this procedure is well established, the validity of the predicted output levels has never been demonstrated clinically. Furthermore, this procedure requires a mobile and visually accessible stapes and an AMEI stimulating the ossicular chain. Thus, an alternative method is needed to quantify the output level of AMEIs in all other stimulation modes, e.g. reverse stimulation of the round window. Intracochlear pressure difference (ICPD) is a good candidate for such a method as it correlates with evoked potentials in animals and it is measurable in cadaveric ears. To validate this method we correlated AMEI output levels calculated from ICPD and from stapes vibration in cadaveric ears with outputs levels determined from clinical data. Output levels calculated from ICPD were similar to output levels calculated from stapes vibration and almost identical to clinical data. Our results demonstrate that both ICPD and stapes vibration can be used as a measure to predict AMEI clinical output levels in cadaveric ears and that ICPD as reference provided even more accurate results.

## Introduction

Implantation of an AMEI is a common treatment for sensorineural or mixed hearing loss. Such a device converts external sound to vibration that stimulates structures of the ossicular chain (e.g. the incus or the stapes), or the round window, a membranous opening to the inner ear (cochlea). In all AMEIs the vibration is produced by an implanted electromechanical transducer.

The output level of the AMEI transducer (actuator) for a given driving voltage affects the gain and maximum loudness that the AMEI system can provide, and is therefore an important factor in determining the range of hearing loss for which the device is considered an appropriate treatment. Current best practice for predicting the output level of an AMEI stimulating the ossicular chain before clinical data are available is defined in the “Standard Practice for Describing System Output of Implantable Middle Ear Hearing Devices” F2504-05^1^, published by ASTM in 2005. It describes a procedure to quantify AMEI output levels as equivalent sound pressure level [eq. dB SPL] by comparing stapes vibration amplitudes measured with a laser Doppler vibrometer (LDV) in human cadaveric temporal bones (TBs, containing the external, middle, and inner ear), in response to sound and to AMEI stimulation. This procedure is based on the finding that the sound transmission through the human middle ear is comparable in cadaveric and live ears^2–4^. Although this method is commonly used^5–8^, it has never been demonstrated, to our knowledge, that output levels predicted from cadaver studies according to ASTM F2504-05 actually match the real outputs in patients. Furthermore, the ASTM procedure has a limited applicability: First, the procedure requires a mobile stapes. Second, stapes vibration is a valid measure of the input to the inner ear only in forward stimulation where the direction of sound transmission is identical to normal hearing and vibration of the stapes is the input to the inner ear^8^. Both is the case only if an implant vibrates the ossicular chain and the cochlea is left intact, but not in other common stimulation modes^9–12^ where an AMEI drives the round window or the cochlear fluid is directly stimulated by a direct acoustic cochlear implant (DACI). In a DACI application the stapes footplate is perforated to insert the tip of an actuator into the cochlea fluid. The DACI converts acoustical signals into vibrations to stimulate the cochlear fluid mechanically. If the round window is excited by an actuator, the ear is stimulated in reverse direction and the acoustic input impedance is different compared to the input impedance during acoustic stimulation, leading to an underestimation of the real stimulation output if stapes vibration is used^8^. In direct stimulation of the cochlear fluid with a DACI the stapes footplate is perforated and bypassed, making stapes vibration measurements meaningless. To estimate the output level of such stimulation in cadaveric TBs, the vibration responses of the round window is commonly measured by an LDV instead^13,14^. However, perforating the stapes footplate and opening the cochlea changes the acoustic input impedance of the inner ear and causes a strong change in the round window motion pattern above 1.5 kHz^15^. Therefore round window vibration measurements are unreliable to quantify the stimulation output of a DACI. A potential alternative method to quantify in cadaver studies the output level of an AMEI or DACI in all stimulation modes is the measurement of the difference in sound pressure between scala vestibuli (SV) and scala tympani (ST)^16^, two fluid filled canals in the cochlea. The so called intra cochlea pressure difference (ICPD) has been shown to correlate with auditory evoked potentials in animals^17^, has been successfully measured during forward and reverse stimulation in TB experiments^8,16,18–20^, is considered as the input to the cochlea^19^ and is measurable with off-the-shelf sensors^16^.

The aim of this study was twofold. First, to test if the procedure predicting clinical equivalent actuator output of an AMEI from cadaver experiments according to ASTM F2504-05 is valid. Second, to validate that ICPD can be used to predict the output level of an AMEI from cadaver studies. For this purpose, AMEI equivalent actuator output levels (eq. dB SPL) in cadaveric ears determined from stapes vibration amplitudes and from ICPD amplitudes were for the first time directly compared to equivalent actuator output levels obtained for the same actuator type and stimulation mode from clinical data.

## Materials and Methods

The equivalent sound pressure level (eq. SPL) produced by an AMEI actuator (T2 transducer, Cochlear™ Ltd.) stimulating the incus body was quantified in human cadaveric TBs using both stapes motion and ICPD as a reference. This stimulation mode was chosen because stapes vibration measurement according to ASTM 2504-05^1^ is the recommended measure of cochlear excitation in this application. Additionally, results from cadaveric ears were compared with T2 actuator output levels obtained from clinical data. Most of the methods used here were similar to previous studies^13,16^ containing more detailed descriptions of the experimental setup.

### Temporal bone preparation

Fourteen already frozen, anonymized human cadaveric TBs were obtained from the Institute for Pathology of the Hannover Medical School. All TBs were harvested in autopsies and the donors were anonymous and no biographical donor data are known. Harvesting and use of the TBs was conducted in accordance with the Helsinki declaration and approved by the ethics committee of the Hannover Medical School (approval No. 3452-2016). All TBs were harvested within 48 h *post mortem*, immediately frozen at approx. −19 °C and thawed shortly before preparation. The stapes and the promontory were exposed by a mastoidectomy, a removal of the facial nerve and drilling of the bony rim of the round window niche overhang down to approx. 1 mm was performed. After preparation the TBs were refrozen in saline containing ~0.005‰ thimerosal and thawed shortly before the experiments. During experiments the preparations were moistened with saline to avoid changes in mechanical behavior^1^.

### Experimental setup

TBs were fixed in a laboratory clamp and a custom-made sound application setup comprising a probe microphone (ER-7C, Etymotic Research Inc., USA) and a loudspeaker (DT48, beyerdynamic, Germany) was cemented (Paladur, Heraeus Kulzer GmbH, Germany) into the outer ear canal. The tip of the microphone’s probe tube was positioned 2–3 mm from the tympanic membrane.

A T2 actuator (Cochlear™ Ltd.) as used in the Cochlear™ MET® system or in the Cochlear™ Carina® system was glued (Sekundenkleber blitzschnell, UHU GmbH & Co KG, Germany) to a rod mounted to a force sensor (LSB200, FUTEK Advanced Sensor Technology, USA). This assembly was attached to a 3-axis micromanipulator (M3301R, World Precision Instruments Germany GmbH, Germany) allowing adjustments in all three spatial directions while monitoring the axial force by the force sensor. The entire setup was installed on a vibration isolated table (LW3048B, Newport).

### Vibration measurement

Vibrations of the stapes were recorded with a single-point LDV system (CLV 700, HLV 1000, HLV MM2, Polytec, Germany) either at the footplate or at the posterior crus of the stapes, depending on the visual access in the TB preparation. To increase the reflectance, a small piece (<0.3 mm × 0.3 mm) of retroreflective tape (Polytec, Germany) was placed at the measurement site. The visually estimated angle of incidence of the laser beam was 30° to 45° to the normal of the SFP and was considered during analysis by a cosine correction of the vibration magnitudes.

### Intracochlear pressure measurement

**Figure 1:**
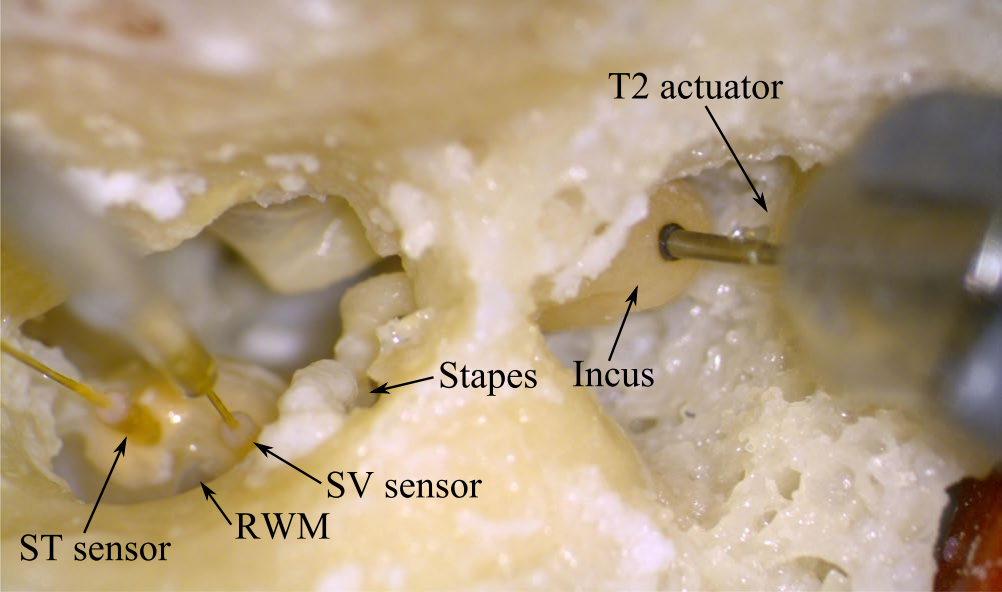
Temporal bone preparation for the incus stimulation. The tip of the T2 actuator was attached to the incus body and the FISO FOP-M260 transducers were inserted in scala vestibuli (SV) and scala tympani (ST) next to the round window membrane (RWM).

The method used for intracochlear sound pressure measurement is described in detail in a previous publication^16^. Here, sound pressure in SV (*P*_*SV*_) and ST (*P*_*ST*_) was recorded simultaneously with fiber-optic pressure transducers with a diameter of 310 µm (FOP-M260, FISO Technologies, Canada) connected to a two-channel signal conditioner (Veloce 50, FISO Technologies, Canada). Transducers were inserted 100–300 µm (visually estimated) into both scalae through fenestrations of approx. 0.4 mm diameter and sealed with a 3 mm piece of silicone tube (Sedat, France) permanently mounted to the optical fiber and dental impression material alginate (Alginoplast®, Heraeus Kulzer GmbH, Germany) (Fig. 1). Phases of the pressure transducers were calibrated in air against a 1/4” reference microphone (Type 4939, Brüel & Kjær, Denmark) and amplitudes against a probe microphone (ER-7C, Etymotic Research Inc., USA). The ICPD (Δ*P*) was calculated as the vector difference of *P*_*SV*_ and *P*_*ST*_ in the frequency domain.

### Experimental procedure

Experiments were performed in TBs having middle ear transfer function within the modified acceptance range^6^ of ASTM standard F2504-05^1^ at 0.25–4 kHz before the insertion of the pressure transducers. Ten out of 14 TBs fulfilled this criterion (Supplementary Figure 1) and were used for the experiments and analysis.

First, the tympanic membrane was stimulated acoustically between 0.1 and 10 kHz with a sequence of 23 pure tones with a frequency resolution of approx. three frequencies/octave and levels of 80–120 dB SPL_TM_ (SPL at the tympanic membrane). For technical limitation of the 4-Ch data acquisition ICPD and vibratory responses were measured sequentially. During the first stimulation the sound pressures *P*_*SV*_ and *P*_*ST*_ were measured by the pressure transducers and during the second stimulation the vibration of the stapes was measured by the LDV. The acoustic input signal at the tympanic membrane was recorded both times by the probe microphone. Although amplitudes and phases of the input signal had minor differences during both stimulations (maximum difference: 0.07 dB, 0.54°), all measurement results were re-normalized to the same input.

After completing the acoustical stimulation, a hole of approx. 0.6 mm was made in the incus for attachment of the actuator using a surgical laser (Iridis, Quantel Medical, France). The actuator stimulation was performed at different contact positions. To define a zero position, the tip of the T2 actuator was advanced towards the laser hole until the tip almost touched the incus and the recording of the force sensor was zeroed. From this position, the actuator was advanced in steps of 20 µm towards the incus body (Fig. 1). At each position, the displayed force level was recorded and actuator stimulation and measurements were performed as follows. The actuator was electrically driven with the same sequence of sine wave signals previously used for the acoustical stimulation, having amplitudes of approx. −8 dB re 1 V_rms_ at each stimulation frequency. Sound pressures *P*_*SV*_ and *P*_*ST*_ and vibration of the stapes were measured sequentially as before. Again amplitude and phase of the input signal had minor differences during both measurements (maximum difference: 0.07 dB, 0.54°) and measurement results were re-normalized to the same input.

From each experiment we had a set of measurement data at different positions and force levels. For this publication we selected the measurement data from two specific forces/positions (see next paragraph for details): (1) The position where the contact force between actuator and incus was closest to 4 mN and (2) the position where the actuator was advanced additional 60 µm from position 1 (hereinafter referred to as “4 mN + 60 µm” position). On average, the contact force was 3.6 ± 2.6 mN in position “4 mN” and 40.0 ± 11.0 mN in position “4 mN + 60 µm” (mean ± standard deviation, N = 10).

After completing all measurements, the pressure transducers were removed and the correct positioning of the cochleostomies in SV and ST and the integrity of the basilar membrane were confirmed visually by dissection of the TB.

### Choosing the contact force / position of the actuator in TB experiments

Actuator positions “4 mN” and “4 mN + 60 µm” were chosen for analysis based on bench tests using the Carina® transducer loading assistant (TLA, Cochlear™ Ltd.). Intraoperatively, the TLA is used to guide adjustment of the T2 actuator to the incus body by measuring its electrical impedance at the resonance frequency while the actuator is advanced^21^. According to the surgical manual^21^ a decrease in impedance of ≥50 Ω indicates initial contact and then the actuator shall be advanced 62.5 µm (1/4 turn of the micro-adjustment) to the final position. In the bench test the actuator was mounted to the force sensor and micromanipulator as in the TB experiments and moved towards a flexible plastic element in steps of 20 µm. In six tests the drop in impedance (initial contact) occurred between 1 and 7 mN (3.4 ± 2.6 mN, mean ± standard deviation). Therefore we selected from the data set of each TB experiment that position where the contact force was closest to 4 mN. To mimic the assumed final position of the loading procedure with the TLA (1/4 turn of the micro-adjustment after indication of the TLA), we selected from each TB experiment also that position where the actuator was moved 60 µm towards the incus from the “4 mN position”.

### Signal generation and acquisition

Signals were generated and acquired with a custom built LabVIEW program controlling two 24 bit, 4-channel data acquisition modules (NI USB-4431, National Instruments, Germany). Electric input signals to the loudspeaker and to the actuator were generated at 25.6 kHz sample rate and buffered by a power amplifier (SA1, Tucker-Davis Technologies, USA). Electric output signals from probe microphone, LDV and FISO pressure measurement system were acquired as averaged complex spectra using 800 Fast Fourier Transformation (FFT) lines between 0 and 10 kHz with 12.5 Hz resolution. During measurement the signal-to-noise ratio (SNR) of the intracochlear pressure and vibratory responses at each stimulation frequency was calculated in LabVIEW using the average of the three adjacent FFT lines below and above as noise level estimate. Measurements were averaged until an SNR of ≥12 dB was reached, but minimally 30 times and maximally 1000 times. In a great majority of measurements an SNR of 20 to 60 dB was already reached with 30 averages and averaging more than 30 times was necessary in a few cases only. Responses with SNR < 12 dB (after 1000 averages) were not considered for analysis.

### Equivalent sound pressure level calculation

Here, actuator output is characterized as “equivalent sound pressure level (SPL)” according to ASTM standard F2504-05^1^, i.e. as the free field sound pressure level required to produce the same stapes vibration or the same intracochlear pressure difference as actuator stimulation with an input voltage of *E*_*max*_.

Equivalent SPLs were calculated from stapes motion as detailed in Grossöhmichen *et al*.^13^. In brief, the unimplanted stapes displacement *d*_*U*_ (calculated from velocity measured by the LDV) in response to ear canal sound pressure *p*_*T*_ at the tympanic membrane was compared to the stapes displacement *d*_*A*_ generated by the actuator attached to the incus driven by input voltage *E*. Based on this data, the maximum achievable equivalent ear canal SPL (*L*_*Emax*_) generated by the actuator at hypothetical input voltage *E*_*max*_ = 1V_rms_ can be calculated as

1 $${L}_{{E}_{{\rm{\max }}}}=20\,{\mathrm{log}}_{10}(\frac{{d}_{A}}{E}\cdot \frac{{p}_{T}}{{d}_{U}}\cdot {E}_{{\rm{\max }}}/(2\cdot {10}^{-5}{\rm{Pa}})).$$

Equivalent SPLs were calculated from ICPD in a similar way. First, the equivalent ear canal sound pressure transfer function *H*_*ET*_ was calculated by comparing the sound-induced ICPD (Δ*p*_*U*_) with the ICPD (Δ*p*_*A*_) generated by the actuator:

2 $${H}_{ET}=\frac{{\rm{\Delta }}{p}_{A}}{E}\cdot \frac{{p}_{T}}{{\rm{\Delta }}{p}_{U}},$$

with *E* being the actuator input voltage and *p*_*T*_ the ear canal sound pressure at the tympanic membrane during acoustic stimulation. With an hypothetical electrical actuator input of *E*_*max*_ *= *1V_rms_, the maximum eq. ear canal SPL at the tympanic membrane (*L*_*Emax*_) was then calculated as

3 $${L}_{{E}_{{\rm{\max }}}}=20\,{\mathrm{log}}_{10}({H}_{ET}\cdot {E}_{{\rm{\max }}}/(2\cdot {10}^{-5}{\rm{Pa}})).$$

Finally, all eq. ear canal SPLs *L*_*Emax*_ [eq. dB SPL_TM_] calculated from stapes vibration (equation (1)) and from ICPD (equation (3)) were converted to eq. free field SPLs *L*_*FF*_ [eq. dB SPL_FF_]:

4 $${L}_{FF}={L}_{{E}_{{\rm{\max }}}}-{T}_{d}.$$

*T*_*d*_ [dB] is a frequency-specific sound pressure transformation value given in tables I to III in Shaw *et al*.^22^. At frequencies where no transformation value *T*_*d*_ was given in Shaw *et al*., *T*_*d*_ was estimated by a linear interpolation between the transformation values at the adjacent frequency above and below.

### Clinical data collection

In clinical routine, bone conduction thresholds were measured in 24 recipients of a MET® middle ear implant system coupled to the incus at audiometric frequencies 0.25-6 kHz using conventional equipment. “Direct thresholds” for stimulation via the Cochlear™ MET® middle ear implant system were measured in the same patients using the Cochlear™ Button® Audio Processor as a signal generator, via the Cochlear™ Carina® Fitting Software. Using the fitting software, psychophysical pure tone thresholds can be determined that are displayed in units of “dB MET”, which can be converted to Volts electrical input. We assume that the loudness perception at bone conduction (BC) threshold and at direct threshold is the same. If a recipient has *b* dB HL BC threshold and *a* dB MET direct threshold at the same frequency, we converted *b* to *b′* [dB SPL_FF_] using ANSI S3.6-2004^23^ Table 9, and *a* to *a′* [dB V] using information from Jenkins *et al*.^24^. Actuator output *L*_*E*_ [eq. dB SPL_FF_] at 1 V_rms_ input voltage was then calculated as:

5 $${L}_{E}=b^{\prime} -a^{\prime} .$$

In rare cases where the BC threshold was not measurable at a particular frequency and no indication for an air-bone-gap (ABG, difference between air conduction and bone conduction hearing thresholds) was visible in preoperative results (ABG ≤ 10 dB), the postoperative corresponding BC threshold was estimated from the measured air conduction threshold.

### Data availability

All data analyzed during this study are included in this published article and its Supplementary Information files.

## Results

As described in method section “Equivalent sound pressure level calculation”, all actuator output levels are expressed as equivalent free field sound pressure level [eq. dB SPL_FF_], calculated by comparing stapes vibration amplitudes and ICPDs measured in response to sound and to actuator stimulation according to equations (1) to (4). In other words, actuator output [eq. dB SPL_FF_] presented here is the free field sound pressure level needed to produce a stapes vibration amplitude or ICPD amplitude equal to that produced by the actuator stimulation. All clinically and experimentally determined actuator output levels presented here were normalized to a hypothetical actuator input voltage of 1 V_rms_. Actuator output levels determined experimentally are shown for an actuator position with a coupling force of approx. 4 mN and for a position where the actuator was moved additional 60 µm towards the incus. The former corresponds to a static force level found experimentally for the point of initial contact for the suggested loading procedure with the TLA and the latter corresponds to the final position of the loading procedure with the TLA recommended by the manufacturer (see materials and methods section for details).

### Actuator output calculated from stapes motion

#### 4 mN static coupling force

**Figure 2:**
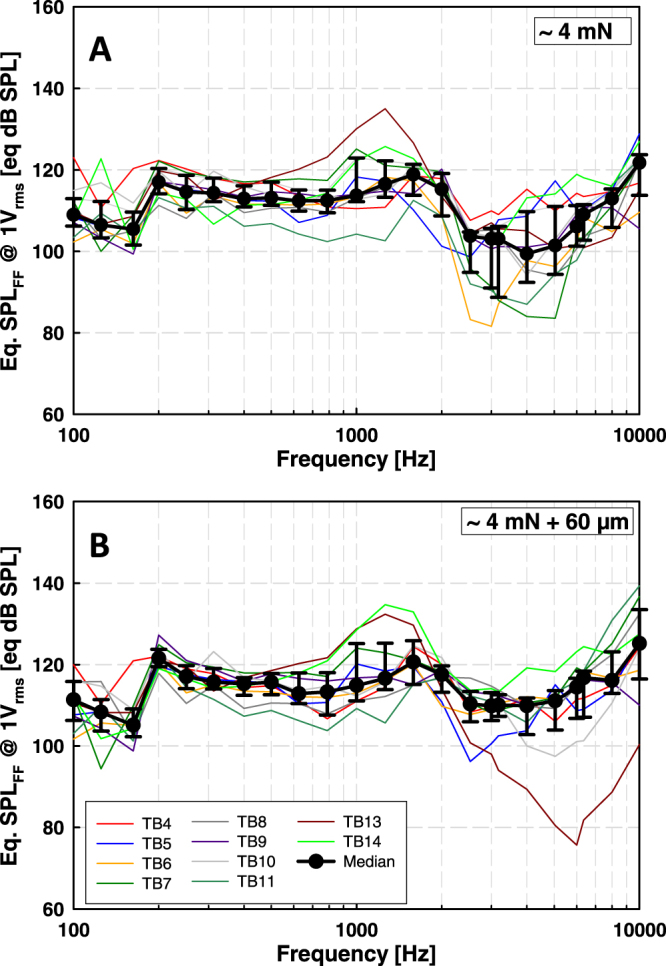
Actuator output (eq. dB SPL_FF_) in TBs, calculated from stapes vibration amplitudes. Colored thin lines represent results from individual TBs, black circles medians, and error bars 25% and 75% percentiles. (**A**) Results obtained at ~4 mN static coupling force. (**B**) Results obtained when the actuator was advanced 60 µm from position A.

Based on stapes vibration amplitude, the actuator produced in the individual TBs between 82 and 135 eq. dB SPL_FF_ (Fig. 2A). The results were normally distributed (Shapiro-Wilk test) at all frequencies except at 3.175 kHz (p = 0.030). The median output level was in the range of 100 to 122 eq. dB SPL_FF_.

#### 4 mN + 60 µm

Actuator output in individual TBs, calculated from stapes vibration amplitude, was mostly in the range of 95 to 140 eq. dB SPL_FF_ (Fig. 2B) with the exception of TB13 where it was 75 to 90 eq. dB SPL_FF_ between 4 and 8 kHz. Actuator output level was normally distributed (Shapiro-Wilk test) at all frequencies except at 3.175 kHz (p = 0.008), 5.0375 kHz (p = 0.012), 6 kHz (p = 0.006) and 6.35 kHz (p = 0.015). The median output level was between 105 and 125 eq. dB SPL_FF._

### Actuator output calculated from ICPD

#### 4 mN static coupling force

**Figure 3:**
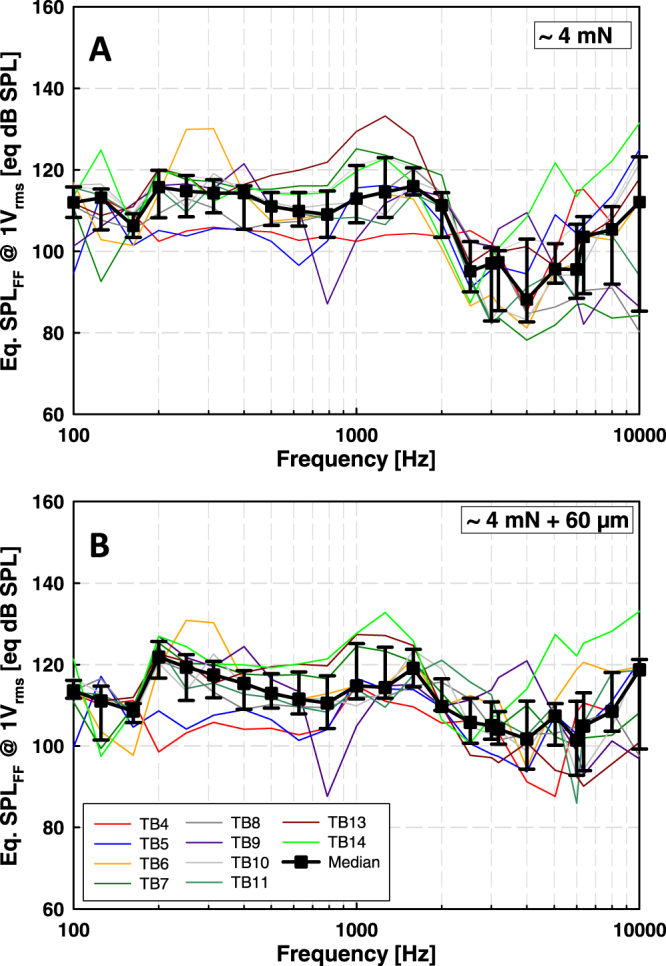
Actuator output (eq. dB SPL_FF_) in TBs, calculated from intracochlear pressure differences. Colored thin lines represent results from individual TBs, black squares medians, and error bars 25% and 75% percentiles. (**A**) Results obtained at ~4 mN static coupling force. B: Results obtained when the actuator was advanced 60 µm from position A. Data points having SNR < 12 dB was omitted (A: TB04 at 10 kHz and TB09 at 0.1 and 0.1625 kHz; (**B**) TB04 at 10 kHz, TB09 at 0.1 to 0.1625 kHz and TB13 at 8 kHz).

At frequencies ≤ 2 kHz, the actuator output calculated from ICPD in the individual TBs was mostly in the range of 100 to 133 eq. dB SPL_FF_ (Fig. 3A). Only at some frequencies in TB5, TB07 and TB09, the output was lower. Above 2 kHz the individual results showed an increased inter-individual variability with outputs from 78 to 131 eq. dB SPL_FF_. Responses in TB04 at 10 kHz and in TB09 at 0.125 & 0.1625 kHz had an SNR < 12 dB and were excluded from analysis. The results were normally distributed (Shapiro-Wilk test) at all frequencies except 0.1 kHz (p = 0.017) and 3 kHz (p = 0.023). The median output levels were in the range of 88 to 116 eq. dB SPL_FF_.

#### 4 mN + 60 µm

Based on ICPD, actuator output in individual TBs was 86 to 133 eq. dB SPL_FF_ over the entire frequency range (Fig. 3B). Responses in TB04 at 10 kHz, in TB09 at 0.1 to 0.1625 kHz and in TB13 at 8 kHz had an SNR < 12 dB and were excluded from analysis. Outputs were normally distributed (Shapiro-Wilk test) at all frequencies except at 0.2 kHz (p = 0.011) and 1.2625 kHz (p = 0.044). The median output levels were in the range of 102 to 122 eq. dB SPL_FF_.

### Actuator output determined from clinical data

**Figure 4:**
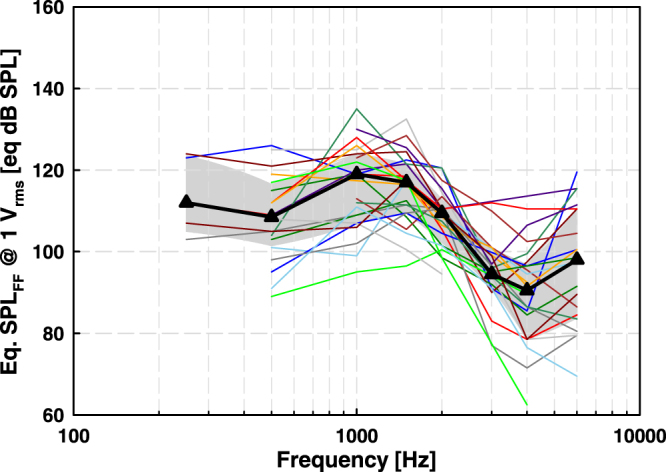
Actuator output (eq. dB SPL_FF_) in patients calculated from direct and bone conduction thresholds. Colored thin lines depict results from individual patients, black triangles median and the grey shaded area 25% and 75% percentiles. Number of patients contributing data at 0.25 kHz (N = 5), 0.5 kHz (N = 20), 1 kHz (N = 23), 1.5 kHz (N = 24), 2 kHz (N = 24), 3 kHz (N = 16), 4 kHz (N = 22) and 6 kHz (N = 20).

Actuator output levels (eq. SPL_FF_) in patients were calculated from BC thresholds and direct thresholds measured with the fitting software using the sound processor as a signal generator (see materials and methods section). Output levels in individual patients were between 90 and 135 eq. dB SPL_FF_ at 0.25 to 2 kHz and mainly between 80 and 120 eq. dB SPL_FF_ at 3 to 6 kHz (Fig. 4). The median eq. SPLs were in the range of 90 to 120 eq. dB SPL_FF_. The results were normally distributed (Shapiro-Wilk test) at all frequencies. Data points are missing at frequencies where the direct or bone conduction threshold was not or could not be measured.

### Actuator output in cadaveric ears: stapes vibration vs. ICPD

#### 4 mN static coupling force

**Figure 5:**
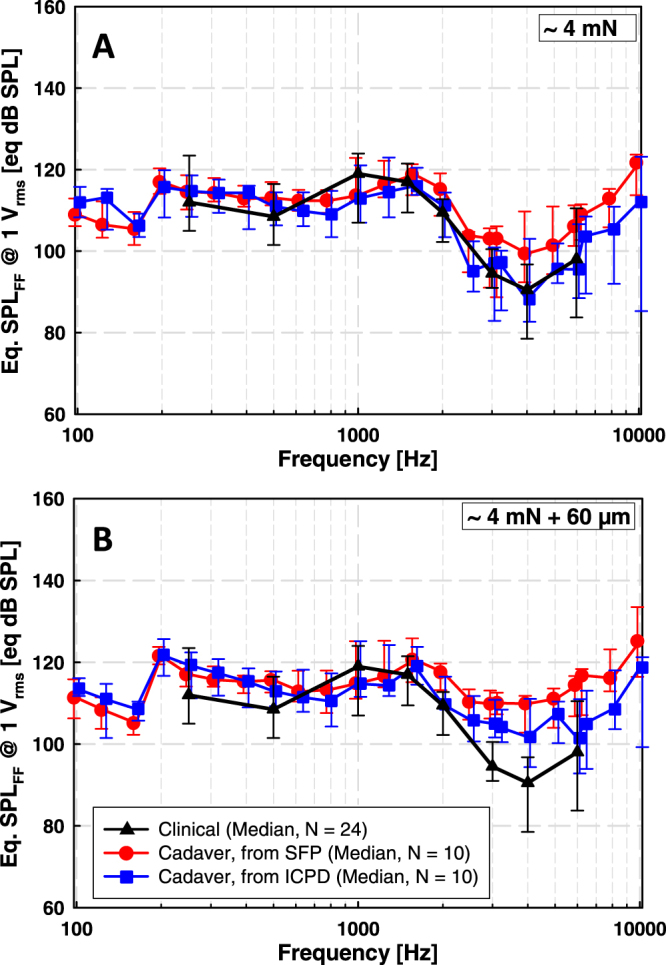
Comparison of median actuator output levels (eq. dB SPL_FF_) in cadaveric TBs, calculated from ICPD (blue), stapes motion (red) and based on clinically measured psychoacoustic thresholds (black). Error bars represent 25% and 75% percentiles. Blue and red symbols and error bars are shifted by ±2% along the frequency-axis for clarity. (**A**) Cadaver results obtained at ~4 mN static coupling force. (**B**) Cadaver results obtained when the actuator was advanced 60 µm from position A.

Median eq. SPLs calculated from stapes motion and ICPD were in good accordance with differences of <4 dB at frequencies below 2 kHz (except 7 dB at 0.125 kHz) and of 4 to 11 dB at higher frequencies (Fig. 5A). The difference between the actuator outputs calculated from stapes motion and ICPD was statistically not significant (Wilcoxon Signed Rank Test), except at 0.625 kHz (p = 0.049), 0.7875 kHz (p = 0.049), 2.525 kHz (p = 0.037), 3 kHz (p = 0.027), 3.175 kHz (p = 0.014), 4 kHz (p = 0.037), 5.0375 kHz (p = 0.049), 6 kHz (p = 0.006) and 8 kHz (p = 0.049).

#### 4 mN + 60 µm

Median eq. SPLs calculated from stapes motion and from ICPD were similar with differences of < 4 dB at frequencies below 2 kHz and of 4 to 13 dB at higher frequencies (Fig. 5B). At most frequencies the differences were statistically not significant (Wilcoxon Signed Rank Test), except at 2 kHz (p = 0.027), 3.175 kHz (p = 0.049) and 8 kHz (p = 0.027).

### Actuator output in cadaveric ears vs. clinical data

Actuator outputs measured in TBs and in patients were statistically compared at audiometric frequencies 0.25, 0.5, 1, 1.5, 2, 3, 4 and 6 kHz. Actuator output level in TBs at 1.5 kHz was estimated by a linear interpolation between the measured outputs levels at 1.2625 and 1.5875 kHz.

#### 4 mN static coupling force

The median actuator outputs calculated from stapes vibration amplitudes in TBs were similar to the output in patients with differences of 1 to 6 dB at 0.25 to 2 kHz and of 8 to 9 dB at higher frequencies. The differences were statistically not significant (Mann-Whitney Rank Sum Test), except at 2 kHz (p = 0.047) and 4 kHz (p = 0.027). Median actuator output levels obtained in cadaveric TBs from ICPD and in patients were very similar over the entire frequency range with statistically non-significant (Mann-Whitney Rank Sum Test) differences of 6 dB at 1 kHz and of < 3 dB at all other frequencies (Fig. 5A).

#### 4mN + 60 µm

Median actuator output levels obtained in TBs and clinical results had similar frequency dependency (Fig. 5B). The differences between the median eq. dB SPL_FF_ calculated from stapes vibration amplitudes in TBs and from clinical data were 3 to 8 dB at 0.25 to 2 kHz and 15 to 19 dB at 3 to 6 kHz. Differences were statistically not significant (Mann-Whitney Rank Sum Test) at 0.25 to 1.5 kHz but statistically significant at higher frequencies (p = 0.002 at 2 kHz, p < 0.001 at 3 and 4 kHz, p = 0.021 at 6 kHz). The differences between the median eq. dB SPL_FF_ calculated from ICPD in TBs and from clinical data were ≤7 dB and statistically not significant (Mann-Whitney Rank Sum Test) at 0.25 to 2 kHz and at 6 kHz. At 3 and 4 kHz the differences were 11 dB and statistically significant (p = 0.002 at 3 kHz, p = 0.005 at 4 kHz, Mann-Whitney Rank Sum Test).

## Discussion

### ICPDs were comparable to literature

When normalized to ear canal sound pressure input level *p*_*T*_ at the tympanic membrane, the magnitudes and phases of the ICPD measured during acoustical stimulation were mostly in good accordance with data from literature^19^ (Supplementary Figure 2).

### AMEI output levels calculated from stapes motion were comparable to literature

Using the “classic” reference stapes vibration amplitude, the output of a T2 actuator in incus body stimulation had also been determined in earlier cadaver studies by Tringali *et al*.^25^ and Devèze *et al*.^7^. Output levels in these studies were given as eq. SPLs at the tympanic membrane [eq. dB SPL_TM_] and have been converted^22^ to eq. free field SPLs [eq. dB SPL_FF_] for direct comparison with our results calculated from stapes motion (Supplementary Figure 3). At most frequencies the median output obtained in our study at actuator position “4 mN + 60 µm” was similar to the mean results of both studies. However, in the publication by Tringali *et al*. (5 TBs) the T2 actuator output seems to be overestimated in the mid-frequency range. It may have contributed that 2 out of 5 TBs in Tringali *et al*. had middle ear transfer functions outside the modified acceptance range^6^ of ASTM standard F2504-05^1^ at 0.25–4 kHz. The output in Devèze *et al*. (6 TBs) was similar to our data except for frequencies ≤ 0.6 kHz although coupling forces were not specified in their study. To our knowledge, this is the first study quantifying the eq. SPL output of mechanical stimulation of the ossicular chain in human cadaveric TBs from ICPD measurements. In existing studies^8,18^ with a Floating Mass Transducer (Vibrant MED-EL Hearing Technology GmbH) ICPDs were measured during mechanical stimulation of the round window in TBs, but not converted into eq. SPLs.

### AMEI output levels calculated from ICPD were similar to output levels calculated from stapes vibration according to the “gold standard method” ASTM 2504-05. Deviations at higher frequencies could be due to the complex nature of stapes motion

As incus stimulation is within the scope of ASTM 2504-05, eq. SPL calculated from stapes motion and from ICPD should be similar. In our study, the median actuator output level calculated from stapes motion and from ICPD were almost identical below 2 kHz, independent from the force/position of the actuator (“4 mN” or “4 mN + 60 µm”) and thus from the level of contact force (4 mN or 40 mN). However, at higher frequencies the actuator output level determined from stapes motion was up to 13 dB higher. This discrepancy may be explained by the fact that the complex nature of stapes motion at higher frequencies as rocking motions are more likely to affect stapes vibration measured at a single location than ICPD that integrate pressure fields at a more remote location in the cochlea. In both live and cadaveric human ears the stapes motion is primarily piston-like below 1 kHz, but above this frequency rocking motions become more dominant with frequency^26,27^. Based on numerical simulations, rocking motion does not produce net volume displacement of the perilymph and has negligible effects on cochlear excitation^28^. Thus, rocking motion components measured at a single point with a 1-D single-point LDV may be misinterpreted as piston-like motions and contribute to the measured vibration amplitude. Of course, this would have no effect on the eq. SPL calculation if the complex motion pattern of the stapes were identical in the acoustic reference stimulation and the mechanical stimulation. However, in our study the eq. SPLs estimated from stapes motion differed up to 13 dB from the eq. SPLs estimated from ICPD and obtained from clinical data above 2 kHz. Therefore we assume that, the pattern of the complex stapes motion at high frequencies changed when the T2 actuator vibrated the incus body in a direction different to the direction of incus motion during acoustic stimulation. Under this assumption, the use of stapes vibration amplitudes measured with a 1-D single-point LDV as reference could lead to a slight overestimation of the real stimulation output as it is visible in our results (Fig. 5). In contrast, ICPD considered as the input to the cochlea^19^ is a result of the net volume displacement of the stapes footplate and should not be affected by stapes rocking motions. This assumption is confirmed by our study as the eq. SPL estimates from ICPD and clinical data were almost identical at all frequencies from 0.1 to 10 kHz (Fig. 5A).

### In incus stimulation, both measurements of ICPD and of stapes movement can be used to predict the achievable loudness levels of AMEIs, but ICPD as reference provided even more accurate results

Independent from the reference (stapes motion or ICPD), all estimated eq. SPL were close to the eq. calculated from clinical data at actuator position “4 mN”. However, above 2 kHz the prediction from ICPD was even more accurate. Therefore we assume that ICPD is a better reference for eq. SPL estimation from cadaver studies that is not affected by altered stapes motion patterns at higher frequencies as discussed above.

### Discrepancy between AMEI output levels estimated from cadaver experiments and clinical data could be due to unexpectedly low coupling forces in patients

At the assumed final position (60 µm from the initial contact of 4 mN) for the loading procedure with the TLA, the median actuator output (eq. dB SPL_FF_) determined in our TB experiments from ICPD was similar to the output obtained from clinical data at frequencies ≤ 2 kHz but up to 11 dB higher at frequencies above. At the detection limit of the TLA (static coupling force of approx. 4 mN), however, output levels from cadaver and clinical data were very similar over the entire frequency range. These results suggest that the post-operative coupling force in our patients was also approx. 4 mN. One potential reason for the presumably smaller force level in our patients may be that in our clinic the final contact position of the T2 actuator during surgery is determined not by using the TLA but by measuring the stapes vibration response with an LDV during actuator stimulation. Based on our experience this method is more sensitive than using the TLA, because proper stapes vibration responses are measurable even at loading forces below 4 mN. Another reason might be a long-term relaxation of the ossicular chain in patients leading to a shift of the incus and a decreasing contact force. Long-term effects such as tissue growth around the actuator that may occur in patients could not be simulated in the TB experiments. At least, an attenuating effect of surrounding tissue on the mechanical output of the AMEI actuator is unlikely^29^.

## Conclusion

In this study the achievable loudness of an AMEI stimulating the incus was estimated by measuring stapes velocity and ICPD in human cadaveric ears. These estimates were compared to each other and to audiometric data from patients treated with the same AMEI type and stimulation mode. At 4 mN coupling force, all estimates were very close (maximal difference of 6 dB) up to 3 kHz. Above 3 kHz, the stapes velocity estimate deviated up to 11 dB from ICPD estimate and up to 9 dB from clinical data, whereas the estimate from ICPD and clinical data were almost identical for all frequencies from 0.1 to 10 kHz. This study demonstrates for the first time that both ICPD and stapes motion can be used as a valid measure to predict the clinically achievable loudness of AMEIs in cadaver studies. However, ICPD as reference provided results matching the output from clinical data even better and has the advantage of being applicable to stimulation scenarios where the stapes footplate is not possible as reference.

## Electronic supplementary material

Supplementary Information
