# Supplementary material for: Validation of methods for prediction of clinical output levels of active middle ear implants from measurements in human cadaveric ears
Source: Sci Rep. 2017 Nov 20;7:15877. doi: 10.1038/s41598-017-16107-9 (PMC5696479; doi:10.1038/s41598-017-16107-9)
Supplement: Supplementary file 1 — Supplementary Information [file 41598_2017_16107_MOESM1_ESM.pdf]

Validation of methods for prediction of clinical output levels of active middle ear implants from measurements in human cadaveric ears

-

Supplementary Information

Martin Grossöhmichen[1,2,*], Bernd Waldmann[3], Rolf Salcher[1,2], Nils Prenzler[1,2], Thomas Lenarz[1,2], Hannes Maier[1,2]

[1] Department of Otolaryngology and Institute of Audioneurotechnology (VIANNA), Hannover Medical School, 30625 Hannover, Germany

[2] DFG Cluster of Excellence "Hearing4all", Germany

[3] Cochlear Deutschland GmbH & Co. KG, 30625 Hannover, Germany

[*] Corresponding author: grossoehmichen.martin@mh-hannover.de

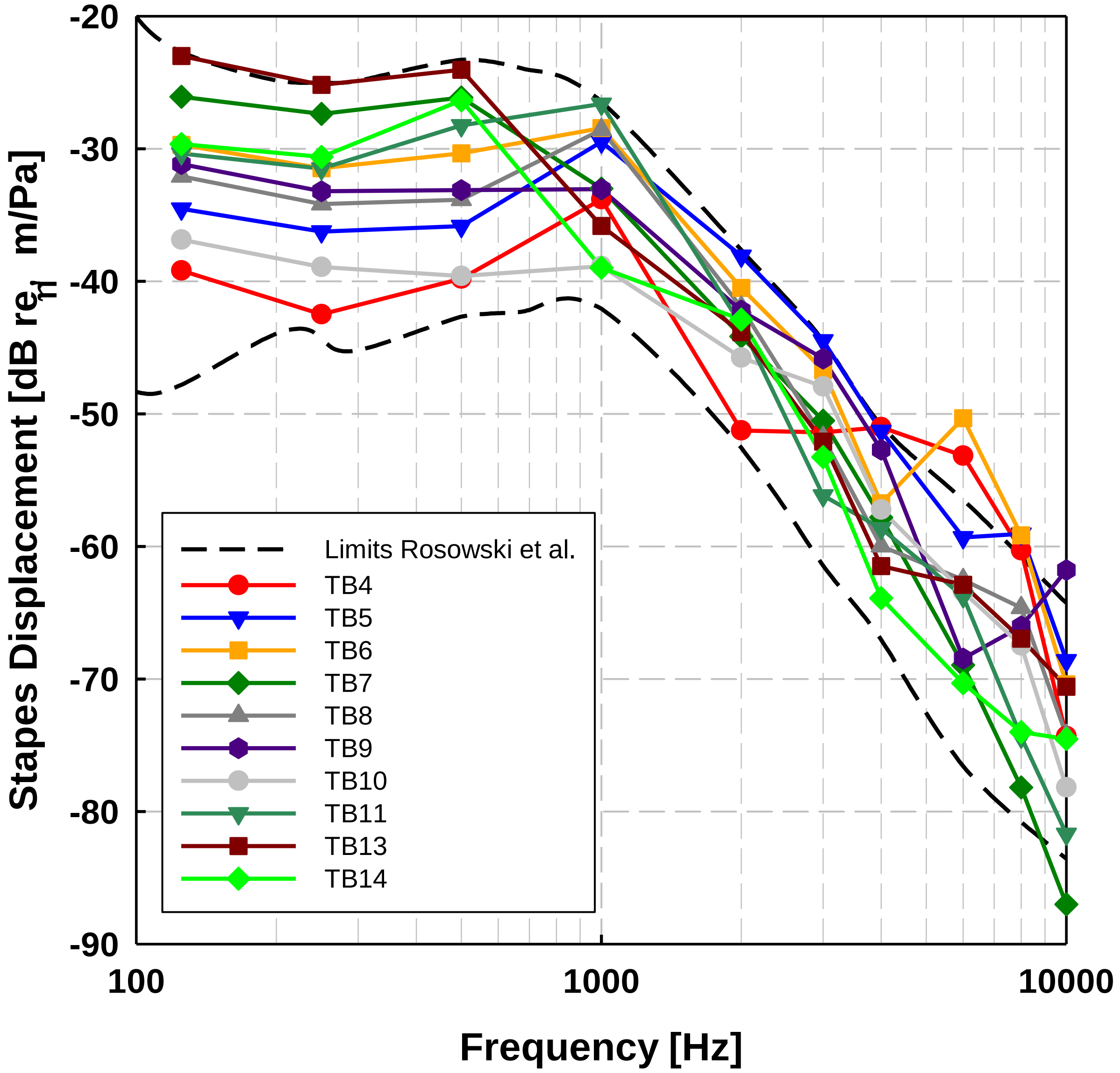

Supplementary Figure 1: Stapes displacement responses to sound stimulation at the tympanic membrane in temporal bone preparations used for experiments. The black dashed lines depict the limits given by Rosowski et al.[3].

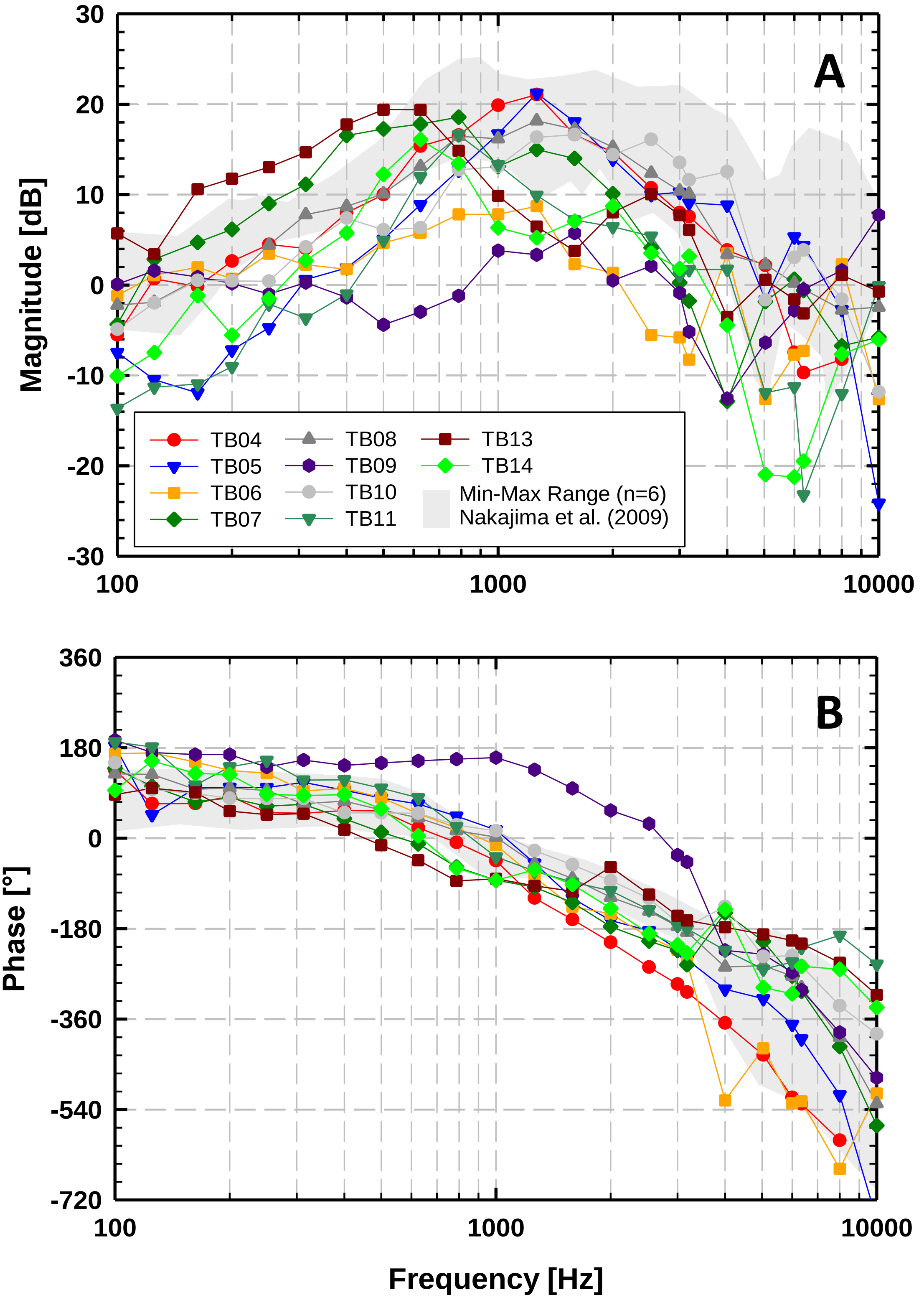

Supplementary Figure 2: Intracochlear pressure differences (ICPD) normalized to the outer ear canal sound pressure level (p_T). For comparison the range of results[16], obtained with a custom made pressure sensor are given as grey shaded area.

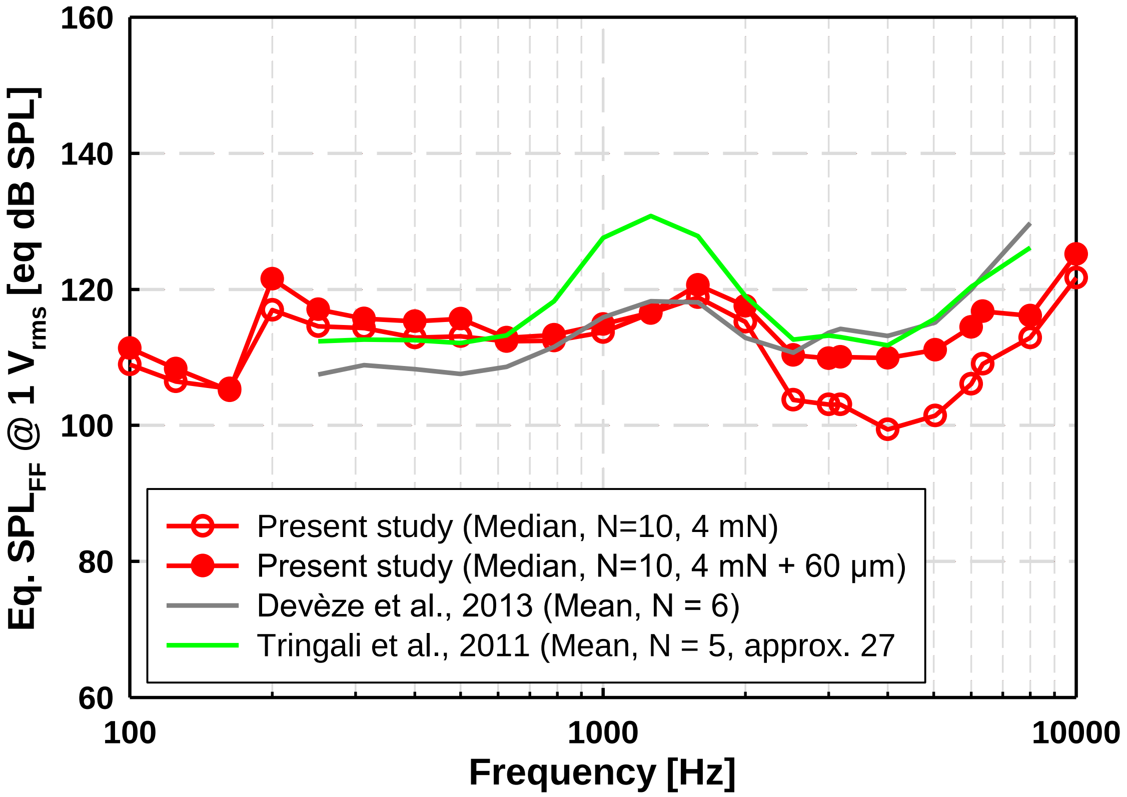

Supplementary Figure 3: Comparison of the T2 actuator output (eq. dB SPL_{FF}) in TBs, calculated from stapes vibration amplitudes, between the present study, Devèze et al.[4] and Tringali et al.[22]. Output levels in Devèze et al. and Tringali et al. were given as eq. ear canal SPLs [eq. dB SPL_{TM}] for nominally 1 V_{rms} actuator input voltage and have been converted to eq. free field SPLs [eq. dB SPL_{FF}] using tables I to III in Shaw et al.[19] to make them comparable to our data.
